# The Role of Friends in Supporting Young People With Cancer: A Scoping Review

**DOI:** 10.1002/pon.70107

**Published:** 2025-02-16

**Authors:** Rebecca L. Sampson, Fiona E. J. McDonald, Vincent O. Mancini, Peter M. McEvoy, Amy L. Finlay‐Jones

**Affiliations:** ^1^ School of Population Health Curtin University Perth Australia; ^2^ Early Neurodevelopment and Mental Health The Kids Research Institute Australia Nedlands Australia; ^3^ Canteen Australia Sydney Australia; ^4^ Faculty of Medicine and Health University of Sydney Sydney Australia; ^5^ Human Development and Community Wellbeing The Kids Research Institute Australia Nedlands Australia; ^6^ Division of Paediatrics UWA Medical School University of Western Australia Perth Australia; ^7^ enAble Institute Curtin University Perth Australia

**Keywords:** adolescent, cancer, friends, neoplasm, psycho‐oncology, review, social support

## Abstract

**Objective:**

Peers play a crucial role in supporting wellbeing and psychosocial development for young people aged 12–25. However, a cancer experience often leads to challenges maintaining friendships. There have been no prior attempts to map or synthesise available research or resources on support specifically from friends for young people with cancer, limiting the capacity to draw conclusions or determine next steps for how to best support young people with cancer. This review aims to address this gap by mapping and synthesising the available literature and resources.

**Methods:**

Included studies were required to obtain data from young people with a history of cancer or their friends, and have a main aim or outcome related to relationships between young people with cancer and their friends. Five databases (Medline, Web of Science, Embase, ProQuest and PsycInfo) were searched, and grey literature were sourced using three search engines (Brave Browser, DuckDuckGo, and Google).

**Results:**

A total of 52 studies and 10 resources met inclusion criteria. Three main themes were identified within the literature (valued friendship dimensions and actions, cancer‐related challenges to friendships, and changes to social needs, experiences, and outcomes). Resources for friends included education about what young people with cancer may experience, advice on supporting or talking to their friend, and encouragement for self‐care.

**Conclusions:**

Reviewed studies highlight the importance of friends support for young people with cancer, and the challenges faced. We present a future research agenda to address identified gaps, including the absence of studies exploring the perspectives and needs of friends.

## 1 Introduction

### 1.1 Cancer in Young People

Worldwide, an estimated 247,754 young people aged 10–24 were diagnosed with cancer in 2022 [1]. While the World Health Organization [2] define adolescents and young adults as those aged 10–24 years, adolescent and young adult oncology has limited consensus on included ages [3]. By any definition, young people with a cancer diagnosis are faced with unique challenges when compared to young children and adults [4, 5], and are vulnerable to greater and more enduring distress [6, 7]. During the period of adolescence and young adulthood, people search for a sense of self and identity [8, 9], begin developing roles they will occupy as an adult, and expand social relationships beyond the family unit to focus more on peers [10]. Chronic conditions such as cancer have been found to change an individual's sense of self and disrupt typical patterns of lifespan development, as their identity requires adjustment to include physical limitations, emotional impacts, and the way one conceptualises their illness [11]. Additionally, gaining independence can be challenging for young people with cancer as difficulties obtaining or maintaining employment can complicate financial independence [4], while physical impacts can impede independence across activities of daily living [12].

### 1.2 Impact of Cancer on Social Functioning and Relationships

Peer relationships are an essential form of social support among young people, helping to promote wellbeing, and providing important context for social and emotional development [13]. Evidence suggests that building effective communication skills, engaging in social activities, and ongoing social support influence positive social relationships, which can act as a protective factor against depression in young people [14]. However, a cancer diagnosis introduces significant challenges for their social functioning and relationships [13], with significantly lower perceived social support than healthy peers [15]. Many young people with cancer report feeling isolated, that they miss out on opportunities their peers are partaking in, and that they struggle to communicate their experience with peers [5]. A study by Kaluarachchi et al. [16] found that friendships can support young people with cancer in maintaining a sense of normalcy by enabling maintenance of their pre‐cancer identity and can offer valued distraction to daily life with cancer. Perceived support from friends has been found to correlate with lower psychological distress (*r* = −0.31, *p* < 0.01), and improved positive affect (*r* = 0.47, *p* < 0.01) [17]. However, friendships can also be challenged when a young person receives a cancer diagnosis and throughout their cancer journey [13]. Young people have reported friends being overly supportive can feel inauthentic, friends progressing with life can serve as a reminder of their situation, and physical impacts of cancer (e.g., fatigue or pain) can make maintaining contact difficult [16].

Research on young people's perspectives found that they felt it is important to have friends who understand what it means to have cancer, and have accurate information [16]. This helps the young person to feel supported and valued, while facilitating maintenance of current friendships [16]. Inversely, when friends lack an accurate understanding about the impact of cancer, young people report negative effects on friendships, such as friends being distant or dismissive [16]. While this research highlights several facets of social support and friendship for young people with cancer, there have been no prior attempts to synthesise this literature. Given the evidence for the importance of friends within this age group and the impact cancer has on friendships for young people, synthesising this area of research will help to guide future research in the field and improve support from friends for young people with cancer.

Accordingly, the primary aim of this scoping review is to identify and integrate what is known about various dimensions of social support among young people with cancer and highlight gaps in the literature. Scoping reviews are a valuable method for determining the breadth of a body of literature, identifying gaps, and developing more specific research questions [18]. Therefore, a scoping review was selected given the range of research in the area and the intention to develop several broad questions for future investigation.

### 1.3 Objectives

The objective of this review is to understand what is known about friendships in young people with cancer, including:

- What aspects of friendships do young people with cancer find most helpful?
- What aspects of friendships are challenging for young people with cancer?
- What resources currently exist to help friends support a friend with cancer and how effective are they?

## 2 Methods

The review was conducted and reported in line with the Preferred Reporting Items for Systematic Reviews and Meta Analyses‐Scoping Review extension for scoping reviews, PRISMA‐ScR [19]. The five general steps taken in a scoping review were followed in line with Arksey and O'Malley [20]: (a) Identifying the research question; (b) Identifying relevant studies; (c) Study selection; (d) Charting the data; and (e) Collating, summarising, and reporting the results. This review was pre‐registered in the Open Science Framework (https://osf.io/cqp3v).

### 2.1 Eligibility Criteria

To be included, studies needed to obtain data from young people with history of a cancer diagnosis or from friends of young people with cancer. Friends included anyone young people chose to talk about when asked about friends or friendships and may or may not have included friends with a cancer diagnosis. For either participant group, an age range of 12–25 at the time of the study was required. While there is limited consensus on the age range for adolescent and young adult cancer [3], the eligibility criteria for this review aligns with the service provision of the primary partner for the study, Canteen Australia [21]. Papers needed to report relationships between young people with cancer and their friends, including needs, experiences, supportive resources, and outcomes as a main aim or outcome. Peer‐reviewed and grey literature from any context, any location, and with any design were included, with the exclusion of reviews, conference abstracts, commentary, and opinion pieces. Publications were required to be in English and no date limit was set.

### 2.2 Procedure

#### 2.2.1 Search Strategy

A systematic search was conducted across Medline, Web of Science, Embase, ProQuest, and PsycInfo. Bramer et al. [22] found that using Embase, MEDLINE, Web of Science Core Collection, and Google Scholar allowed for an overall recall of between 98.3% and 100% in 72% of systematic reviews. With the recommendation of including area specialised databases, ProQuest and PsycInfo were used instead of Google Scholar. The search strategy was formed in collaboration with the research team and a research librarian to optimise the balance of specificity and comprehensiveness. Search terms were words relating to cancer, young people, and friends. Database specific search strategies were devised (see Appendix A). Grey literature was sourced through web searches using browsers that do not tailor search results based on prior search history to provide a more objective search. The search engines used in the current study were Brave Browser, DuckDuckGo, and an advanced Google search using incognito mode.

#### 2.2.2 Database Search and Screening

Final database searching was conducted on 3rd October 2023. Studies extracted from each database were exported into EndNote X9. Deduplication was run using sr‐accelerator automatic de‐duplicator which was manually checked by one researcher (RS). Sources were uploaded to Rayyan [23] where further duplicates were identified and removed. Next, all titles and abstracts were co‐screened, with one reviewer screening all sources (RS) and four co‐screeners each screening a portion (BN, HW, CC, AP). One researcher carried out full text screening on all sources (RS), and 30% were co‐screened (AFJ). Any conflicts were discussed against the inclusion criteria and a third reviewer was included where required (VM). Reference lists of all included studies were checked for additional relevant studies (RS).

#### 2.2.3 Grey Literature Search and Screening

Grey literature searching was conducted on 5th May 2023. Two researchers separately conducted the same search to minimise bias (RS, YYL). Each researcher extracted the first 20 results from each search into an Excel spreadsheet and assessed the titles for relevance. Relevant results were then assessed for inclusion suitability by one researcher (RS).

#### 2.2.4 Data Extraction

Data were extracted into Microsoft Excel from sources that met inclusion criteria. Data extracted included study location, setting and aim, participant demographics, scales used, interview guide questions, and key findings. Additional data extracted for qualitative sources included themes and data collection methods. Measures used and the main findings related to social support were extracted from quantitative studies. Resource author, organisation, purpose, main content, and development input information were extracted for grey literature sources.

#### 2.2.5 Data Coding

Study topics were coded across qualitative and quantitative papers to help group findings into meaningful clusters. Coding was an iterative process, and codes were devised and refined as studies were assessed. Codes were assigned based on the study aims, what was being assessed in interviews or surveys, the themes identified in qualitative studies, and outcomes in quantitative or mixed methods studies. Sources could have more than one code assigned if there were multiple topics considered.

## 3 Results

### 3.1 Identifying Relevant Studies

**FIGURE 1:**
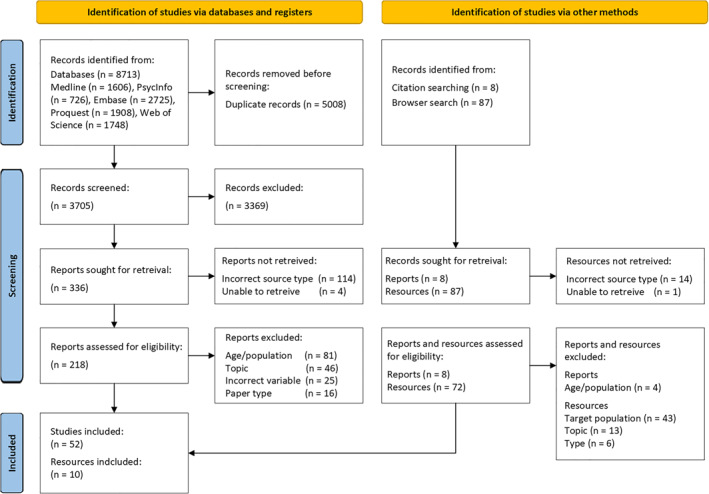
PRISMA 2020 flow diagram for scoping reviews.

A total of 8713 results were returned across the databases (see Figure 1). Initial deduplication reduced the number of sources to 3867. A further 162 duplicates were identified in Rayyan, leaving 3705 sources for title and abstract screening. Reviewers had 94%–96% agreement at the title and abstract screening stage and all conflicts were resolved. In sourcing full text papers, 114 were identified as inappropriate source types (e.g., conference abstracts, dissertations). A total of 218 papers were considered appropriate for full text screening. Following full‐text screening, 49 sources were eligible for inclusion. Reference list screening identified three additional sources for inclusion.

### 3.2 Identifying Relevant Resources

Of the first 20 results on each of the three grey literature search sites, 87 unique results were identified across the two researchers. Of 60 results retrieved by each researcher, 33 (55%) were the same, leaving 27 unique results from each (33 common + 27 unique to researcher one + 27 unique to researcher two = 87 unique results). Before screening, 14 results were excluded as they were journal articles already screened in the systematic review. Additional results were excluded due to not being a suitable source type (*n* = 6), using the incorrect target population (*n* = 43), or having an irrelevant topic (*n* = 13). The remaining 10 sources were retained for data extraction.

### 3.3 Study Characteristics

Key characteristics of the 52 peer‐reviewed studies are reported in Supporting Information: Tables S1–S3. Studies were published between 1984 and 2023 and were conducted in 17 countries, including one study conducted across six countries. The largest proportion of studies was from USA (*n* = 22), followed by Canada (*n* = 7), Australia and the UK (*n* = 5 each), Turkey (*n* = 3), and Denmark, Korea and Sweden (*n* = 2 each). Countries with a single included study are France, India, Israel, Italy, Netherlands, Norway, Poland, Taiwan, and Wales. Across studies, 1718 young people with cancer were included; with 50.5% male, 48.1% female and 0.1% transgender or nonbinary. One source did not provide gender information of the remaining 1.3% of participants [24]. Parents (*n* = 283) provided data in seven studies and were present for interviews in five studies (*n* = 57). Siblings participated in one study (*n* = 15) and two included health care providers (*n* = 12). Two studies included healthy comparison peers (*n* = 320), one included historic data from healthy young people for normative comparison (*n* = 1084) and adults with cancer (*n* = 642), and one study included class peers only (*n* = 65). Critically, none of the studies included friends of young people with cancer. Studies focused on exploring experiences of support for young people with cancer (*n* = 36), evaluating interventions for support from friends for young people with cancer (*n* = 2), assessing relationships between variables (*n* = 7), and understanding support needs before, during, or after a cancer experience (*n* = 7).

Key characteristics of the 10 included grey literature sources can be found in Supporting Information: Table S4. Of the 10 resources for friends of young people with cancer, 50% stated they were written in collaboration with young people with cancer, and 80% were written by organisations specifically providing support to young people with cancer. None provided information to suggest that friends were involved in the resource creation process. Resources were written in the USA (*n* = 4), UK (*n* = 3), and Australia (*n* = 3).

### 3.4 Synthesis of Results

Of the 52 included sources, 38 (73.1%) were qualitative. Of the qualitative studies, a majority collected data using semi‐structured interviews (*n* = 27). The focus of these studies included preferred support strategies [16, 25, 26], challenges with returning to school [27, 28], and benefits of connecting with other young people with cancer [29, 30]. The remaining 14 sources were either quantitative (*n* = 8) or mixed methods (*n* = 6). Some of these studies found relationships between support and distress [17, 31], identified support person preferences [32, 33], and identified differences in social support between young people with cancer and healthy peers [15]. Social support was quantitatively assessed using a range of methods, however, only two scales were used across multiple studies. The Multidimensional Scale of Perceived Social Support [34], which considers three sources of support (family, friends, and significant others), was used across three studies. The Perceived Social Support‐ Friends Scale [35] was used across four studies and includes questions about how an individual feels their friend's support them.

Following coding of all studies, three major themes were identified; valued friendship dimensions and actions; cancer‐related challenges to friendship; and changes to social needs, experiences and outcomes for young people living with cancer.

#### 3.4.1 Valued Friendship Dimensions and Actions

The first theme of valued friendship dimensions and actions identified aspects of friendships and specific actions by friends and cancer peers that were helpful for young people with cancer. This theme was coded in 30 studies (57.7%). Most studies addressing this theme were qualitative (*n* = 26), with 4 mixed methods studies and no quantitative studies. This theme had two main elements.

The first element was dimensions of friendships and actions that were helpful and supportive from friends without a cancer experience. Within this element, studies (*n* = 23) identified valued dimensions of friendships with healthy peers, and why they were helpful. For example, David et al. [36] found that young people with cancer valued friendships that helped them maintain a sense of normality. Other studies reported that friendships provide a sense of safety or protection for young people when they have cancer [25, 37]. Some studies highlighted that young people felt it was important to have a best friend [27, 38] or a group of friends that genuinely cared to make it easier to return to normality [27]. Helpful aspects of friendships included having company [26, 39, 40] or emotional support [38], such as friends visiting in hospital and making an effort to maintain contact [13]. Helpful forms of support from friends allowed adolescents to feel included, normal and cared for [16].

The second element identified in 11 studies was that other young people with cancer were identified as a helpful source of support. A common reason identified for why these cancer peer relationships were valuable was around having a unique and deep understanding from a shared experience [16, 41]. Young people with cancer valued friendships with cancer peers as they provided a relationship within which to be genuine about fears and to joke about their cancer [16, 42]. Young people also found a sense of empathy and hope about the future [43]. These friendships with peers allowed young people with cancer to feel less isolated [16, 44].

#### 3.4.2 Cancer‐Related Challenges to Friendships

The second theme reflected the challenges to friendships that young people face when they have a cancer diagnosis. This was coded in 17 studies (32.7%), with 16 being qualitative, and one other study, which considered difficulties with friends and social networks, using a mixed methods approach [33]. Four main elements were identified within this theme: unhelpful interactions with friends, stigma and loss of normality, challenges with disclosure and communication, and difficulties discerning true friends.

The first element of this theme, identified in 13 studies, considered unhelpful interactions with friends, characteristics of these interactions, and specific unhelpful behaviours. Kaluarachchi et al. [16] found that some young people felt that being treated differently or awkwardly by friends was unhelpful, and other studies reported young people experienced their friends distancing themselves [45, 46]. Unhelpful actions identified across studies included friends being absent during treatment [26], excessively publicising support efforts [16], and making negative comments about changed appearance [45]. Some unhelpful attempts at support by friends left young people feeling more concerned about their condition, invalidated, or as though the support was not genuine [16]. Some young people felt their friends were uncomfortable talking about cancer, which led to the young person feeling that their friend did not care enough to support them [40]. Unique difficulties of having cancer peers were also identified in three studies, with some young people expressing heightened concern about their condition upon knowing someone with the same illness [42] and others feeling reluctant to form friendships with cancer peers to avoid potential grief if they died [16].

The second element, reported across 12 studies was that young people commonly experienced stigma from other young people, felt a sense that they had lost normality of being a young person [47], and reported feeling isolated from friends [45, 48]. Some studies reported that young people felt they lacked support due to not feeling understood [33], or that friends did not understand the impact of cancer [16]. Examples of feeling a loss of normality included young people describing friends treating them differently, assuming they could not participate in activities or providing uncomfortable excessive attention [16, 48]. Some young people described difficulty feeling normal on return to study with unwanted attention due to their appearance [49] or that their study accommodation signalled being “different” to peers resulting in insensitive comments [48]. Some young people reported a sense of disconnect with their previous friends group on return to school and feeling like they did not “fit in”, often attributed to the young person feeling they had changed due to their cancer experience [28, 47].

The third element across studies (*n* = 6) was disclosure and communication. For example, young people in some studies found it difficult to decide when and how to tell new friends about their cancer diagnosis [50]. Other studies identified that some young people felt worried they might cause their friends discomfort by talking about their cancer, which led to difficulty initiating conversations about their condition [27] or feeling that they needed to portray they were coping well to avoid discomfort [50]. Kaluarachchi et al. [16] identified that discomfort was experienced as feeling dismissed for some young people as they felt their friends' avoided discussions about cancer or understated the seriousness after treatment. It was also identified across a number of studies that when young people did talk about their cancer, they felt it was difficult for their friends to understand or know how to react [27, 33].

The fourth element was discerning genuine friends. This element was identified in five studies. Some young people with cancer felt others were overly supportive, and some felt frustrated with sympathetic responses or unwanted attention from peers who did not previously interact with them [50, 51], both of which felt disingenuous [16]. Friendships became less stable for some young people due to being away from usual social activities or feeling too unwell to engage with friends [13, 52], with some studies finding that young people reported a sense of acceptance with losing friends as their more genuine friends were identified through their illness [53].

#### 3.4.3 Changes to Social Needs, Experiences, and Outcomes

The final theme was the social needs, experiences, and outcomes that were identified as changing across the cancer experience for young people. This theme also considered the complexities of social adjustment combined with the transition from adolescence to adulthood. Social needs, experiences, and outcomes was the most frequently identified theme across all study types, being coded in 29 studies (55.8%). Of the studies coded in this theme, 17 were qualitative, eight quantitative, and four mixed methods.

The first element of this theme, reported across eight studies, was young people identified new social needs throughout the cancer experience, including needs that were not relevant when they were healthy. For example, many young people found a need to continue feeling a sense of normalcy [16, 30]. Friends helped fulfil this need by adapting conversations to focus less on the young person and cancer‐related topics and more on the everyday life of the healthy friend [30, 54]. This also helped fulfil a newly identified need to be distracted from daily life with cancer [55]. Young people with cancer also felt that friends played an important role in shielding them from others' negative opinions and judgement about changed appearance [25, 37, 49]. Additionally, navigating friendships while unwell was difficult as young people had a new need to balance looking after their health by avoiding crowded social settings or friends that were unwell, while staying connected to friends and receiving support [56]. Young people felt they needed to find a way to advocate for themselves, which was a new challenge.

The second element of this theme, reported across 15 studies was around changing social experiences during cancer, including the ways friendships changed. Some young people felt misunderstood by their friends [57] and struggled to connect with cancer patients of other ages [33], leaving them to often feel closest to other young people with cancer [16]. This was highlighted with reports of a strong bond within cancer peer friendships [30]. Multiple studies identified that navigating friendships while unwell was difficult and at times young people felt they could not engage in typical activities with their friends [46, 53]. These experiences aligned with a sense that friendship ties became more tenuous following time away for treatment [26], and some young people felt a sense of alienation following the cancer experience [28, 48]. In contrast, a number of studies reported that when young people felt well supported and cared for by friends during their cancer treatment, friendships strengthened as a result of the shared experience [13, 16, 52].

The third element of this theme related to changes in social outcomes, coded in seven of the included studies. Most quantitative and mixed methods studies highlighted the positive social outcomes related to having strong friendships. For example, when support from friends was rated higher, distress was lower [17] and post‐traumatic growth was better [17, 58]. Perceived social support was found to be significantly lower in young people with cancer than healthy peers [15], and lowest in young people with cancer who had lower self‐perceived social functioning [59].

#### 3.4.4 Support Resources for Friends

Of the 10 resources for friends of young people with cancer that were identified via the grey literature search, five stated they were written in collaboration with young people with cancer. None of the sources provided information to suggest that friends were involved in the resource creation process. All included support resources provided friends with information to gain a better understanding of what their friend with cancer might be experiencing physically and emotionally, and some included information about possible cancer treatments. Some specific ideas of how to support a friend with cancer were included across resources. Specific ideas included visiting a young person with cancer in hospital (*n* = 5), continuing to invite the young person places (*n* = 4), and keeping in touch with messages (*n* = 7). Four resources suggested creating a personalised care package. Giving a gift was suggested by four resources and ideas included comfortable clothes [60, 61], a book [62], and easy activities for them to enjoy [60, 63]. Another common inclusion was what to say to a friend with cancer (*n* = 9) and what to avoid saying (*n* = 6). Most resources also encouraged friends to ensure they were engaging in self‐care and sourcing their own support (*n* = 9). These ideas included friends talking about their own feelings [64, 65], talking to a trusted adult such as a teacher or parent [62, 66], a counsellor or other professional [60, 61, 62, 66, 67]. One source provided a step‐by‐step guide for how to create a support network for the young person who has cancer [67]. This was the only resource that included specific steps of how to provide ongoing support from a range of sources for a friend with cancer.

## 4 Discussion

In this review, we sought to identify and synthesise available research on the area of friendships and social support for young people with cancer, and identify available resources to help friends support a young person with cancer. We found a large body of primarily qualitative research that focussed on the experiences and perspectives of young people with cancer but no studies that focussed on the experiences or perspectives of their friends. Most studies were descriptive (*n* = 49) with only three quasi‐experimental studies evaluating support. Across 52 included studies, we identified consistent findings and commonalities, which we categorised into three main themes [1]: valued friendship dimensions and actions [2]; cancer‐related challenges to friendships; and [3] changes to social needs, experiences, and outcomes for young people with cancer. All 10 resources for friends were found in the grey literature search and were aimed at helping friends of young people with cancer understand the cancer experience better. No evaluations of the impacts of resources were identified in the literature. This review highlights a lack of evidence for the needs and experiences of friends of young people with cancer, absence of the involvement of friends in creating support resources and evaluation, and inconsistency in measuring social support.

A key finding of this review was the aspects of friendships and specific actions friends carry out that young people with cancer value. These helpful and supportive actions allowed young people to feel included and distracted from cancer‐related challenges, which helped with maintaining friendships. Specific actions identified through the literature were supported by the recommendations made in the included resources. The literature also highlighted social challenges faced by young people with cancer including unhelpful actions from friends, a loss of normality in friendships, and difficulty discussing their experience with friends. Understanding these preferences is important for providing specific, clear, and accurate support advice to friends. By synthesising these key findings, we can help improve the chance of young people maintaining their essential social supports, in turn, promoting wellbeing and avoiding delays in social and emotional development [13]. Findings in similar populations show some congruent themes and a similar level of understanding. For example, we found resources for friends of young people with cancer were limited to psychoeducation booklets. Literature has documented that siblings of young people with cancer also experience limited formal support beyond psychoeducation [68]. Additionally, we found that young people with cancer desire connection with cancer peers as they experience a sense of acceptance within these peer relationships [30]. Adults experiencing cancer reported a similar sense of connection and acceptance within peer support groups, which contrasts with the sense of isolation and lack of understanding from healthy friends [69]. Comparing the findings among these groups highlights areas where evidence around support from friends is at a similar level.

In contrast to areas where research is at a similar level to other populations impacted by cancer, significant gaps were also evident in the research for support for young people with cancer. This included the absence of research considering friend's perspectives or needs for providing support confidently. This is an important gap which has been addressed in breast cancer populations. Research has examined how friends can provide better support, which has yielded a detailed and nuanced understanding of barriers to friends providing support and to patients receiving support [70]. Therefore, further investigation is warranted to understand how friends could be better prepared to support young people with cancer. Support resources found within this study also lack friends' perspectives in their development, and therefore may be inadequate. Identified resources, primarily websites and electronic documents, offered limited skill‐building recommendations and suggested support for friends, which may leave friends feeling isolated when they are unsure how to provide support. A broader range of resources for friends of people with cancer exist, including videos and support groups, however, they are not specific to young people with cancer and therefore may not be relevant to their unique needs. For example, information on helping with a friend's children, cooking, house cleaning, and being a carer may not be relevant. This highlights that a more tailored range of resources is needed for this age group. The development of resources should also include the perspectives and experiences of friends, and be more engaging.

No consistent or specific measure of social support from friends was used across the quantitative literature. Previous reviews on social support for young people with cancer have recognised this inconsistency and highlighted the difficulty in reliably making comparisons about the experiences of social support across studies [71, 72]. The only scales used across multiple studies were The Multidimensional Scale of Perceived Social Support [34] and The Perceived Social Support—Friends Scale [35]. These measures are not specific to young people, the context of cancer, or the unique challenges young people with cancer face. The scales used were very broad in the questions asked, types of support measured, and scale purpose. For example, many studies used quality of life scales to measure support for young people with cancer, which provides limited information on the impact of support. A more specific scale for measuring support from friends in young people impacted by cancer, for example, the Cancer Peer Support Scale [73], would allow a better understanding of this experience and allow for more direct comparisons across studies. Measures assessing the quality of social support and friendships may also be valuable in future research.

### 4.1 Implications

Given the overall scope of included research, it can be suggested that young people with cancer likely experience barriers to receiving effective support from friends. While some specific ideas of how to help, what to say, what might be appreciated, and some challenges are presented, it is difficult to identify the most relevant or useful support young people with cancer desire or need from friends. Additionally, given that the needs of friends have not been explored, practical challenges may exist for friends of young people with cancer in confidently providing meaningful support. These gaps may contribute to a lack of effective resources to help friends build their supportive communication skills. In combination, these gaps along with the importance of friendships within this age group [13], are likely to perpetuate poor support and mental health outcomes for young people with cancer. By synthesising available literature and resources, it is hoped the findings presented can contribute to finding the most relevant target and direction of future resources and research. In turn, this may better position friends to effectively support young people living with cancer.

### 4.2 Limitations

Our review has some limitations that should be taken into account when considering implications and directions for future research. Firstly, only studies written in English were included, which may result in a lack of diversity of perspectives and experiences. Second, there were no intervention studies included in this review. This may have been due to limiting inclusion criteria, for example, in some studies, outcomes or themes relating to support from friends were unable to be distinguished from support from family or other sources. This may also indicate support intervention studies may not focus solely on friends for social support. While this evidence could contribute to understanding support for young people with cancer, this review aimed to focus specifically on support from friends given the importance of this type of support within the age group. Time since diagnosis and treatment status of young people with cancer were not assessed in this review. As such, studies in this review include three groups of young people: those in treatment, survivors of childhood cancer who are now young people, and survivors of cancer. These groups potentially have unique support needs which have not been specifically identified. While efforts were made to minimise bias in the grey literature search, it is not possible to eliminate bias in web searching. Additionally, given the nature of the web, the scope of results that could practically be screened was limited, which may exclude resources that exist.

### 4.3 Future Directions

Guidance for future directions for research can be drawn from studies of young people with other chronic conditions. For example, quantitative research with young people with Cystic Fibrosis helped identify and rate specific behaviours that were supportive or unsupportive [74]. Such studies are a useful next step for youth cancer research, and findings could provide clear guidance for resource development. Additionally, understanding when and how frequently young people would like to be supported by friends has not been identified and may be considered in future research. The impact of time since diagnosis and treatment stage may be an additional consideration for further research to analyse as it may impact the way young people with cancer want to be supported. Future research should also focus on gaining a better understanding of the needs of friends from their perspective. This may include what friends want to know to improve their confidence in supporting a young person with cancer, how and when they would like information delivered, and potential ongoing support they may require. This would increase friends' confidence in supporting a young person with cancer and allow young people with cancer to be effectively supported by friends.

## 5 Conclusions

This review provides a holistic view of what is known within the area of friendships and support from friends for young people with cancer. The gaps in the area have been highlighted, and recommendations for what may be needed next are provided. Together, this will allow for better informed recommendations for future research into the friendship support needs of young people with cancer, and what friends within the population might need to improve their support skills. Future research may wish to explore specific support young people need from their friends and how this would be best delivered. As highlighted by other reviews in the area, using more specific measures for social support would help to overcome limitations in synthesising findings [71, 72]. Additionally, exploring what friends need to better support young people with cancer should be a priority.

## Supporting information

Supporting Information S1

Supporting Information S2

## Data Availability

Data sharing is not applicable to this article as no new data were created or analysed in this study.

## References

[1] J. Ferlay , M. Ervik , F. Lam , et al., Global Cancer Observatory: Cancer Today (version 1.1) (Lyon, France: International Agency for Research on Cancer, 2022), https://gco.iarc.fr/today.

[2] World Health Organization . Adolescent and Young Adult Health (2023), https://www.who.int/news‐room/fact‐sheets/detail/adolescents‐health‐risks‐and‐solutions.

[3] S. Aubin , R. Barr , P. Rogers , et al., “What Should the Age Range Be for AYA Oncology,” Journal of Adolescent and Young Adult Oncology 1, no. 1 (2011): 3–10: [Editorial], 10.1089/jayao.2011.1505. 26812562

[4] R. Barr , A. Ferrari , L. Ries , J. Whelan , and W. Bleyer , “Cancer in Adolescents and Young Adults: A Narrative Review of the Current Status and a View of the Future,” JAMA Pediatrics 170, no. 5 (2016): 495–501, 10.1001/jamapediatrics.2015.4689. 26999630

[5] B. J. Zebrack , “Psychological, Social, and Behavioral Issues for Young Adults With Cancer,” Cancer 117, no. S10 (2011): 2289–2294, 10.1002/cncr.26056. 21523748

[6] U. Sansom‐Daly , C. E. Wakefield , R. A. Bryant , et al., “Online Group‐Based Cognitive‐Behavioural Therapy for Adolescents and Young Adults After Cancer Treatment: A Multicenter Randomised Controlled Trial of Recapture Life‐AYA,” BMC Cancer 12, no. 1 (2012): 339, 10.1186/1471-2407-12-339. 22862906 PMC3503656

[7] P. Patterson , N. M. D’Agostino , F. E. J. McDonald , et al., “Screening for Distress and Needs: Findings From a Multinational Validation of the Adolescent and Young Adult Psycho‐Oncology Screening Tool With Newly Diagnosed Patients,” Psycho‐Oncology 30, no. 11 (2021): 1849–1858, 10.1002/pon.5757. 34160847 PMC9291177

[8] E. H. Erikson , Identity: Youth and Crisis (Norton & Co, 1968).

[9] B. Kim , P. Patterson , and K. White , “Developmental Considerations of Young People With Cancer Transitioning to Adulthood,” European Journal of Cancer Care 27, no. 6 (2018): e12836, 10.1111/ecc.12836. 29542833

[10] R. Blum , N. M. Astone , M. R. Decker , and V. C. Mouli , “A Conceptual Framework for Early Adolescence: A Platform for Research,” International Journal of Adolescent Medicine and Health 26, no. 3 (2014): 321–331, 10.1515/ijamh-2013-0327. 24486726 PMC4476282

[11] K. Charmaz , “Studying the Experience of Chronic Illness through Grounded Theory,” in New Directions in the Sociology of Chronic and Disabling Conditions, eds. G. Scambler and S. Scambler (London: Palgrave Macmillan, 2010).

[12] D. Gaser , C. Peters , M. Götte , et al., “Analysis of Self‐Reported Activities of Daily Living, Motor Performance and Physical Activity Among Children and Adolescents With Cancer: Baseline Data From a Randomised Controlled Trial Assessed Shortly After Diagnosis of Leukaemia or Non‐hodgkin Lymphoma,” European Journal of Cancer Care 31, no. 2 (2022): e13559, 10.1111/ecc.13559. 35150025

[13] K. M. Fladeboe , A. J. Walker , A. R. Rosenberg , and L. F. Katz , “Relationships Between Adolescents With Cancer and Healthy Peers: A Qualitative Study,” Journal of Adolescent and Young Adult Oncology 10, no. 5 (2021): 555–561, 10.1089/jayao.2020.0133. 33164615

[14] K. Filia , O. Eastwood , S. Herniman , and P. Badcock , “Facilitating Improvements in Young People's Social Relationships to Prevent or Treat Depression: A Review of Empirically Supported Interventions,” Translational Psychiatry 11, no. 1 (2021): 305, 10.1038/s41398-021-01406-7. 34021113 PMC8139977

[15] M. Tremolada , S. Bonichini , G. Basso , and M. Pillon , “Perceived Social Support and Health‐Related Quality of Life in AYA Cancer Survivors and Controls,” Psycho‐Oncology 25, no. 12 (2016): 1408–1417, 10.1002/pon.4072. 26810123

[16] T. Kaluarachchi , F. McDonald , P. Patterson , and T. R. Newton‐John , “Being a Teenager and Cancer Patient: What Do Adolescents and Young Adults With Cancer Find Valuable and Challenging With Their Friends and Cancer Peers,” Journal of Psychosocial Oncology 38, no. 2 (2020): 195–209, 10.1080/07347332.2019.1672847. 31608828

[17] J. S. Kay , V. Juth , R. C. Silver , and L. S. Sender , “Support and Conflict in Relationships and Psychological Health in Adolescents and Young Adults With Cancer,” Journal of Health Psychology 24, no. 4 (2019): 502–517, 10.1177/1359105316676629. 28810372 PMC5812832

[18] Z. Munn , M. D. J. Peters , C. Stern , C. Tufanaru , A. McArthur , and E. Aromataris , “Systematic Review or Scoping Review? Guidance for Authors When Choosing Between a Systematic or Scoping Review Approach,” BMC Medical Research Methodology 18, no. 1 (2018): 143, 10.1186/s12874-018-0611-x. 30453902 PMC6245623

[19] A. C. Tricco , E. Lillie , W. Zarin , et al., “PRISMA Extension for Scoping Reviews (PRISMA‐ScR): Checklist and Explanation,” Annals of Internal Medicine 169, no. 7 (2018): 467–473, 10.7326/M18-0850. 30178033

[20] H. Arksey and L. O'Malley , “Scoping Studies: Towards a Methodological Framework,” International Journal of Social Research Methodology 8, no. 1 (2005): 19–32, 10.1080/1364557032000119616.

[21] Canteen Australia . A Guide to Canteen (2022), https://www.canteen.org.au/sites/default/files/2022‐12/A_guide_to_Canteen_2022.pdf.

[22] W. Bramer , M. Rethlefsen , J. Kleijnen , and O. Franco Duran , “Optimal Database Combinations for Literature Searches in Systematic Reviews: A Prospective Exploratory Study,” Systematic Reviews 6, no. 1 (2017): 245, 10.1186/s13643-017-0644-y. 29208034 PMC5718002

[23] M. Ouzzani , H. Hammady , Z. Fedorowicz , and A. Elmagarmid , “Rayyan‐a Web and Mobile App for Systematic Reviews,” Systematic Reviews 5, no. 1 (2016): 210, 10.1186/s13643-016-0384-4. 27919275 PMC5139140

[24] M. H. Hodges , J. Graham‐Pole , and M. L. Fong , “Attitudes, Knowledge, and Behaviors of School Peers of Adolescent Cancer Patients,” Journal of Psychosocial Oncology 2, no. 2 (1984): 37–46, 10.1300/J077v02n02_03.

[25] S. Larouche and L. Chin‐Peuckert , “Changes in Body Image Experienced by Adolescents With Cancer,” Journal of Pediatric Oncology Nursing 23, no. 4 (2006): 200–209, 10.1177/1043454206289756. 16766685

[26] G. A. McDonnell , E. Shuk , and J. S. Ford , “A Qualitative Study of Adolescent and Young Adult Cancer Survivors' Perceptions of Family and Peer Support,” Journal of Health Psychology 25, no. 5 (2020): 713–726, 10.1177/1359105318769366. 29687735 PMC6167207

[27] A. Choquette , J. E. Rennick , and V. Lee , “Back to School After Cancer Treatment: Making Sense of the Adolescent Experience,” Cancer Nursing 39, no. 5 (2016): 393–401, 10.1097/NCC.0000000000000301. 26390072

[28] J. K. McLoone , C. E. Wakefield , P. Butow , C. Fleming , and R. J. Cohn , “Returning to School After Adolescent Cancer: A Qualitative Examination of Australian Survivors' and Their Families' Perspectives,” Journal of Adolescent and Young Adult Oncology 1, no. 2 (2011): 87–94, 10.1089/jayao.2011.0006. 26812630

[29] Z. K. Papp , B. Somogyi , C. Wilson , and S. Torok , “Health Accpetance Through Camp: Mixed‐Method Data From a Central‐European Therapeutic Recreational Based Camp for Seriously Ill Children,” European Journal of Mental Health 16, no. 2 (2021): 120–145, 10.5708/EJMH.16.2021.2.6.

[30] E. Donovan , S. R. Martin , L. C. Seidman , et al., “The Role of Social Media in Providing Support From Friends for Adolescent and Young Adult (AYA) Patients and Survivors of Sarcoma: Perspectives of AYA, Parents, and Providers,” Journal of Adolescent and Young Adult Oncology 10, no. 6 (2021): 720–725, 10.1089/jayao.2020.0200. 33844938 PMC8742252

[31] S. Manne and D. Miller , “Social Support, Social Conflict, and Adjustment Among Adolescents With Cancer,” Journal of Pediatric Psychology 23, no. 2 (1998): 121–130, 10.1093/jpepsy/23.2.121. 9585638

[32] J. Dunsmore and S. Quine , “Information, Support, and Decision‐Making Needs and Preferences of Adolescents With Cancer: Implications for Health Professionals,” Journal of Psychosocial Oncology 13, no. 4 (1995): 39–56, 10.1300/J077V13N04_03.

[33] D. R. Weidman , P. Desmarais , K. Stevens , et al., “Peer Support Needs of Adolescents With Cancer in Pediatrics: A Canadian Mixed Methods Study,” Journal of Adolescent and Young Adult Oncology 11, no. 4 (2022): 433–438, 10.1089/jayao.2021.0122. 34591689

[34] G. D. Zimet , N. W. Dahlem , S. G. Zimet , and G. K. Farley , “The Multidimensional Scale of Perceived Social Support,” Journal of Personality Assessment 52, no. 1 (1988): 30–41, 10.1207/s15327752jpa5201_2.

[35] M. E. Procidano and K. Heller , “Measures of Perceived Social Support From Friends and From Family: Three Validation Studies,” American Journal of Community Psychology 11, no. 1 (1983): 1–24, 10.1007/BF00898416. 6837532

[36] C. David , K. Williamson , and D. Tilsley , “A Small Scale, Qualitative Focus Group to Investigate the Psychosocial Support Needs of Teenage Young Adult Cancer Patients Undergoing Radiotherapy in Wales,” Supportive Care in Cancer 16, no. 4 (2012): 375–379, 10.1016/j.ejon.2011.08.002. 21925950

[37] H. Williamson , D. Harcourt , E. Halliwell , H. Frith , and M. Wallace , “Adolescents' and Parents' Experiences of Managing the Psychosocial Impact of Appearance Change During Cancer Treatment,” Journal of Pediatric Oncology Nursing 27, no. 3 (2010): 168–175, 10.1177/1043454209357923. 20173081

[38] R. McNeil , M. Egsdal , S. Drew , M. C. McCarthy , and S. M. Sawyer , “The Changing Nature of Social Support for Adolescents and Young Adults With Cancer,” European Journal of Oncology Nursing 43 (2019): 101667, 10.1016/j.ejon.2019.09.008. 31586646

[39] K. Schreiner , D. Grossoehme , S. Friebert , J. Baker , J. Needle , and M. Lyon , “Living Life as if I Never Had Cancer": A Study of the Meaning of Living Well in Adolescents and Young Adults Who Have Experienced Cancer,” Pediatric Blood and Cancer 67, no. 10 (2020): e28599, 10.1002/pbc.28599. 32686240 PMC7719590

[40] R. L. Woodgate , “The Importance of Being There: Perspectives of Social Support by Adolescents with,” Cancer. Journal of Pediatric Oncology Nursing 23, no. 3 (2006): 122–134, 10.1177/1043454206287396. 16624888

[41] J. Cassano , K. Nagel , and L. O'Mara , “Talking With Others Who "just Know": Perceptions of Adolescents With Cancer Who Participate in a Teen Group,” Journal of Pediatric Oncology Nursing 25, no. 4 (2008): 193–199, 10.1177/1043454208319972. 18539910

[42] M. E. Hotchkiss , Z. N. Ahmad , and J. S. Ford , “Cancer–Peer Connection in the Context of Adolescent and Young Adult Cancer: A Qualitative Exploration,” Journal of Adolescent and Young Adult Oncology 12, no. 1 (2023): 83–92, 10.1089/jayao.2021.0170. 35384687

[43] S. C. Sodergren , O. Husson , G. E. Rohde , et al., “A Life Put on Pause: An Exploration of the Health‐Related Quality of Life Issues Relevant to Adolescents and Young Adults With Cancer,” Journal of Adolescent and Young Adult Oncology 7, no. 4 (2018): 453–464, 10.1089/jayao.2017.0110. 29565709

[44] J.‐L. Sawyer , F. Mishna , E. Bouffet , M. Saini , and R. Zlotnik‐Shaul , “Bridging the Gap: Exploring the Impact of Hospital Isolation on Peer Relationships Among Children and Adolescents With a Malignant Brain Tumor,” Child and Adolescent Social Work Journal 40, no. 1 (2023): 91–105, 10.1007/s10560-021-00764-x. 34025015 PMC8130807

[45] M. Thavakugathasalingam and J. K. Schwind , “Experience of Childhood Cancer: A Narrative Inquiry,” Journal for Specialists in Pediatric Nursing 27, no. 2 (2022): e12367, 10.1111/jspn.12367. 35005836

[46] H. Cavusoglu , “Problems Related to the Diagnosis and Treatment of Adolescents With Leukemia,” Issues in Comprehensive Pediatric Nursing 23, no. 1 (2000): 15–26, 10.1080/014608600265183. 11011660

[47] S. Pini , P. Gardner , and S. Hugh‐Jones , “How Teenagers Continue School After a Diagnosis of Cancer: Experiences of Young People and Recommendations for Practice,” Future Oncology 12, no. 24 (2016): 2785–2800, 10.2217/fon-2016-0074. 27312743

[48] An H. , Lee, S. , “Difficulty in Returning to School Among Adolescent Leukemia Survivors: A Qualitative Descriptive Study,” European Journal of Oncology Nursing. 38:(2019): 70–75, 10.1016/j.ejon.2018.12.008 30717939

[49] S. R. Daniels , C.‐C. Yang , S. J. Toohey , and V. W. Willard , “Perspectives on Social Media From Adolescents and Young Adults With Cancer,” Journal of Pediatric Oncology Nursing 38, no. 4 (2021): 225–232, 10.1177/1043454221992319. 33729902

[50] S. Pini , S. Hugh‐Jones , L. Shearsmith , and P. Gardner , “'What Are You Crying for? I Don't Even Know You'‐The Experiences of Teenagers Communicating With Their Peers When Returning to School,” European Journal of Oncology Nursing 39 (2019): 28–34, 10.1016/j.ejon.2018.12.010. 30850135

[51] C. G. Lam , K. J. Cohen , and D. L. Roter , “Coping Needs in Adolescents With Cancer: A Participatory Study,” Journal of Adolescent and Young Adult Oncology 2, no. 1 (2013): 10–16, 10.1089/jayao.2012.0011.

[52] A. J. Walker , F. M. Lewis , Y. Lin , E. Zahlis , and A. R. Rosenberg , “Trying to Feel Normal Again: Early Survivorship for Adolescent Cancer Survivors,” Cancer Nursing 42, no. 4 (2019): E11–E21, 10.1097/NCC.0000000000000629. PMC6336529 30024440

[53] K. Stegenga and P. Ward‐Smith , “On Receiving the Diagnosis of Cancer: The Adolescent Perspective,” Journal of Pediatric Oncology Nursing 26, no. 2 (2009): 75–80, 10.1177/1043454208328767. 19190175

[54] H. Reuman , K. Kerr , J. Sidani , et al., “Living in an Online World: Social Media Experiences of Adolescents and Young Adults With Cancer,” Pediatric Blood and Cancer 69, no. 6 (2022): e29666, 10.1002/pbc.29666. 35293691

[55] A. Ishibashi , R. Ueda , Y. Kawano , H. Nakayama , A. Matsuzaki , and T. Matsumura , “How to Improve Resilience in Adolescents With Cancer in Japan,” Journal of Pediatric Oncology Nursing 27, no. 2 (2010): 73–93, 10.1177/1043454209356786. 20176917

[56] K. A. Pyke‐Grimm , L. S. Franck , B. Halpern‐Felsher , R. E. Goldsby , and R. S. Rehm , “Day‐To‐Day Decision Making by Adolescents and Young Adults With Cancer,” Journal of Pediatric Hematology/Oncology Nursing 39, no. 5 (2022): 290–303, 10.1177/27527530211068718. 35538622 PMC9807778

[57] E. Donovan , S. R. Martin , L. C. Seidman , et al., “A Mobile‐Based Mindfulness and Social Support Program for Adolescents and Young Adults With Sarcoma: Development and Pilot Testing,” JMIR mHealth and uHealth 7, no. 3 (2019): e10921, 10.2196/10921. 30882352 PMC6441858

[58] A. Ekim and A. F. Ocakci , “Relationship Between Posttraumatic Growth and Perceived Social Support for Adolescents With Cancer,” Journal of Hospice and Palliative Nursing 17, no. 5 (2015): 450–455, 10.1097/NJH.0000000000000183.

[59] V. W. Willard , R. Tillery , M. L. Gordon , A. Long , and S. Phipps , “Profiles of Perceived Social Functioning in Adolescent and Young Adult Survivors of Childhood Cancer,” Psycho‐Oncology 29, no. 8 (2020): 1288–1295, 10.1002/pon.5417. 32419288 PMC8852343

[60] Ninox Cancer Support Crew . The Shitshow Companion: How to Be a Good Friend During Cancer (2022), https://www.ninoxcsc.com.au/wp‐content/uploads/2022/08/The‐Shitshow‐Companion.pdf.

[61] Young Lives vs Cancer . Be There for Them: What You Should Do When Your Friend Had Cancer (n.d.), https://www.younglivesvscancer.org.uk/life‐with‐cancer/my‐friend‐has‐cancer/.

[62] C. Wood and Canteen Australia . Wait… Did You Say ‘Cancer’: A Guide to Supporting Your Friend When They Have Cancer (2021), https://www.canteen.org.au/sites/default/files/2022‐12/Guide_to_supporting_friend_when_they_have_cancer.pdf.

[63] A. Masso and American Childhood Cancer Organization . Taking Charge: A Cancer Resource for Friends (2011), https://www.acco.org/wp‐content/uploads/2014/12/Taking‐Charge‐Friends.pdf.

[64] Teenage Cancer Trust . My Friend Has Cancer (n.d.), https://www.teenagecancertrust.org/information‐about‐cancer/my‐friend‐has‐cancer.

[65] Nemours Teen Health. My Friend Has Cancer . How Can I Help? (2015), https://kidshealth.org/en/teens/friend‐cancer.html.

[66] Lynda Jackson Macmillan Centre . Young People With a Relative or Friend With Cancer (2023), https://www.ljmc.org/helpful_hints/hhc247_young_people.pdf.

[67] B‐present. Supporter Roadmap . Your Unique Path to Connection and Support After a Cancer Diagnosis (2022), https://b‐present.org/young‐adult‐cancer‐supporter‐roadmap/.

[68] Wawrzynski S. E. , Schaefer M. R. , Schvaneveldt N. , Alderfer M. A. “Social Support and Siblings of Children With Cancer: A Scoping Review,”Psycho‐Oncology. 30, no. (8) (2021): 1232–1245, 10.1002/pon.5689 33851490 PMC8363579

[69] J. Ussher , L. Kirsten , P. Butow , and M. Sandoval , “What Do Cancer Support Groups Provide Which Other Supportive Relationships Do Not? the Experience of Peer Support Groups for People With Cancer,” Social Science & Medicine 62, no. 10 (2006): 2565–2576, 10.1016/j.socscimed.2005.10.034. 16303220

[70] M. Skeels , K. Unruh , C. Powell , and W. Pratt , “Catalyzing Social Support for Breast Cancer Patients,” Conference on Human Factors in Computing Systems ‐ Proceedings 1 (2010): 173–182, 10.1145/1753326.1753353. PMC3108040 21654894

[71] C. L. Decker , “Social Support and Adolescent Cancer Survivors: A Review of the Literature,” Psycho‐Oncology 16, no. 1 (2007): 1–11, 10.1002/pon.1073. 16917852

[72] A. Deegan , C. Brennan , P. Gallagher , V. Lambert , and S. Dunne , “Social Support and Childhood Cancer Survivors: A Systematic Review (2006–2022),” Psycho‐Oncology 32, no. 6 (2023): 819–833, 10.1002/pon.6128. 36944590

[73] P. Patterson , F. McDonald , R. Tindle , E. Kelly‐Dalgety , B. Zebrack , and D. Costa , “The Development and Preliminary Evaluation of the Cancer Peersupport Scale in Adolescents Living With Cancer,” Psycho‐Oncology 27, no. 12 (2018): 2865–2868, 10.1002/pon.4869. 30156748

[74] D. Barker , K. Driscoll , A. Modi , M. Light , and A. Quittner , “Supporting Cystic Fibrosis Disease Management During Adolescence: The Role of Family and Friends,” Child: Care, Health and Development 38, no. 4 (2012): 497–504, 10.1111/j.1365-2214.2011.01286.x. 21771002 PMC3479957

