# Supplementary material for: The Role of Friends in Supporting Young People With Cancer: A Scoping Review
Source: Psychooncology. 2025 Feb 16;34(2):e70107. doi: 10.1002/pon.70107 (PMC11830860; doi:10.1002/pon.70107)
Supplement: Supplementary file 1 — Supporting Information S1 [file PON-34-e70107-s002.docx]

**Appendix A**

Medline search strategy

|  |  | Medline | Results |
| --- | --- | --- | --- |
| 1 | cancer* or neoplasm* or tumor* or tumour* or leukaemia or lymphoma (title or abstract) | ((cancer* or neoplasm* or tumor* or tumour* or leukaemia* or Leukemia* or sarcoma* or lymphoma*) adj8 (teen* or youth* or adolescent* or young person* or young people or young adult* or child* or paediatric* or pediatric*)).ti,ab. | 102,880 |
| 2 | friend* or peer* or companion* | (friend* or peer* or companion*).ti,ab. | 234,940 |
| 3 | 1 AND 2 | 1 AND 2 | 1360 |
| 4 | Limit to English only |  | 1319 |
