# Supplementary material for: The Role of Friends in Supporting Young People With Cancer: A Scoping Review
Source: Psychooncology. 2025 Feb 16;34(2):e70107. doi: 10.1002/pon.70107 (PMC11830860; doi:10.1002/pon.70107)
Supplement: Supplementary file 2 — Supporting Information S2 [file PON-34-e70107-s001.docx]

# Table 1

## Characteristics of qualitative studies

| **First Author (Year), Country** | **Population** | | **Study topic** | **Data collection** | **Themes** | **Code** |
| --- | --- | --- | --- | --- | --- | --- |
|  | **Age range** | **Sample size** |  |  |  |  |
| An and Lee (2019), Korea | 14-22 | 14 | Challenges fitting in when returning to school. | Semi structured interviews | Feelings of alienation from friends: *difficulty in getting along with friends.* Stuck being different from others: *burdened by excessive care of others.* | Challenges of friendship and support  Changes to social needs, experiences, and outcomes |
| Arpaci et al. (2022), Turkey | 12-19 | 16 | Support needs of young people after cancer treatment. | Semi structured interviews | Expectations: *Support needs* *(peer relations).* | Cancer-related challenges to friendships  Changes to social needs, experiences, and outcomes |
| Barton (2023), USA | 14-25 | 32 | Needs and experiences of young people who have advanced cancer. | Semi structured interviews | I felt very alone: *Being away from family and friends.* | Changes to social needs, experiences, and outcomes |
| Cassano (2008), Canada | 14-20 | 11 | Reflections of the benefits and challenges of peer support groups. | Semi structured interviews and field notes | Satisfying elements of the teen group: *Talking to others who "just know”.* | Valued friendship dimensions and actions |
| Cavusoglu (2000), Turkey | 13-18 | 30 | Problems faced by young people with cancer. | Semi structured interviews | Relations with peers and family (altered after cancer). | Changes to social needs, experiences, and outcomes |
| Cheng et al. (2016), Taiwan | 12-17 | 11 | Types of support received from peers. | Semi structured interviews | Roots for resilience: *receiving support from significant others and religion.* Transformation and growth: *restructuring the relationship with peers.* | Valued friendship dimensions and actions |
| Choquette (2016), Canada | 13-17 | 11 | Reintegrating to school. | Semi structured interviews | Bridging two worlds: *struggling to rebuild friendships, having a best buddy, the positive nature of ongoing friendships.* | Valued friendship dimensions and actions  Cancer-related challenges to friendships |
| Daniels et al. (2021), USA | 15-20 | 7 | Support from friends online around feeling comfortable with appearance. | Semi structured interviews | Attitudes about body image and peer support *(perception of peer judgements and support when posting self-images through treatment).* | Valued friendship dimensions and actions |
| David (2012), Wales | 20-24 | 4 | Ways friends supported or struggled to support young people. | Focus groups | Extrinsic stressors: *peer support.* Intrinsic stressors: *group cohesion.* | Valued friendship dimensions and actions  Changes to social needs, experiences, and outcomes |
| Donovan et al. (2021), USA | 14-23 | 10 | Benefits of connecting with cancer peers. | Semi structured interviews | Social media facilitates the need to be understood by peers who have experienced sarcoma. | Changes to social needs, experiences, and outcomes |
| Enskär and Berterö (2010), Sweden | 20-23 | 7 | Long term effects of cancer treatment for young people. | Semi structured interviews | Friendship and support from others nearby. | Valued friendship dimensions and actions |
| Fladeboe et al. (2021), USA | 12-17 | 14 | Acts from friends that make young people feel supported or isolated. | Semi structured interviews | Shifting relationships: *becoming more distant, bringing us closer, finding my real friends.* Staying connected: *keeping up with communication.* Making it hard to stay close: *being far away, making them uncomfortable.* Showing me they care: *being there for me, checking in, giving me gifts.* | Valued friendship dimensions and actions  Cancer-related challenges to friendships  Changes to social needs, experiences, and outcomes |
| Hotchkiss et al. (2022), USA | 16-24 | 26 | Experiences with cancer peers. | Semi structured interviews | Unique component of shared illness experience: *specialising of support from personal experience, space to be scared and joke about cancer.* Benefits for those providing support: *giving back.* | Valued friendship dimensions and actions |
| Ingersgaard et al. (2021), Denmark | 14-19 | 14 | Experience of support from a peer-ambassador to re-enter school. | Semi structured interviews | Ambassadors as ‘behind the scenes’ friends. | Valued friendship dimensions and actions |
| Kaluarachchi (2020), Australia | 16-22 | 12 | Valuable support types and challenges experienced with friends. | Semi structured interviews | Valued with friends: *support and understanding, closer relationships.* Challenging with friends: *difficulty in friendships, overly supportive, changing relationships.* Valued with peers: *shared cancer experience.* | Valued friendship dimensions and actions  Cancer-related challenges to friendships  Changes to social needs, experiences, and outcomes |
| Lam et al. (2013), USA | 13-19 | 16 | Friends helping young people cope. | Focus groups (guided by semi-structured questions) | Needs in coping with cancer: *social perceptions (altered).* | Cancer-related challenges to friendships  Changes to social needs, experiences, and outcomes |
| Larouche (2006), Canada | 14-17 | 5 | Support from friends around body image. | Semi structured interviews | Impact of body image perception on their life: *avoiding social situations.* Maintaining normality: *peer-shield.* | Valued friendship dimensions and actions  Cancer-related challenges to friendships |
| McDonnell et al. (2020), USA | 16-24 | 26 | Ways friends provided support or a lack of support. | Semi structured interviews | Support: *practical support, navigating school, emotional support, spending time together, new sense of closeness and appreciation.* Lack of support: *absence during treatment.* | Valued friendship dimensions and actions  Cancer-related challenges to friendships  Changes to social needs, experiences, and outcomes |
| McLoone et al. (2011), Australia | 12-20 | 19 | Challenges of returning to school. | Semi structured interviews | Social experiences *(as a barrier or enabler to school re-entry).* | Cancer-related challenges to friendships |
| McNeil et al. (2019), Australia | 15-25* | 60 | Support from healthy peers and cancer peers. | Semi structured interviews | Providers of social support: *healthy peers, cancer peers.* | Valued friendship dimensions and actions |
| Oh (2019), South Korea | 15-21 | 7 | Acceptance or challenges feeling the same as friends. | Semi structured interviews | Attributes of social adjustment in adolescent cancer survivors: *having harmonious relationships with friends.* | Cancer-related challenges to friendships  Changes to social needs, experiences, and outcomes |
| Olsen and Harder (2009), Denmark | 15-22 | 12 | Perceptions of a network-focused program. | In-depth interviews and observations | Embracing the program: *pulling together.* | Valued friendship dimensions and actions |
| Pini et al. (2016), UK | 13-17 | 12 | Support from friends in returning to school. | Photo elicitation interviews | Changing peer dynamics and norms. | Valued friendship dimensions and actions  Changes to social needs, experiences, and outcomes |
| Pini et al. (2019), UK | 13-16* | 12 | Friends support in school return and disclosure. | Photo elicitation interviews | Approaches to telling, lives becoming public property, owning the story. | Cancer-related challenges to friendships |
| Pyke-Grimm et al. (2022), USA | 15-20 | 16 | Daily decision making by YP with cancer and navigating relationships. | Semi structured interviews | Self-advocacy: *preventing illness or complications.* Navigating relationships: *relationships with friends.* | Changes to social needs, experiences, and outcomes |
| Reuman et al. (2022), USA | 12-25 | 39 | Experiences of cancer related social media use. | Online survey (open and closed ended questions) | Positive experiences of social media use: *support.* | Valued friendship dimensions and actions |
| Ritchie (2001), USA | 12-18 | 45 | Ways friends support young person with cancer. | Open ended questions with written responses | Friends identified as social support person. | Valued friendship dimensions and actions |
| Salins et al. (2023), India | 12-19 | 28 | Understanding the support from friends for young people with cancer. | Semi structured interviews | Coping family, friends and self: *peer support.* | Valued friendship dimensions and actions |
| Sariman (2022), Australia | 18-25 | 10 | Friends during the cancer experience as a young person living regionally. | Semi structured interviews | Connection with people: *family, friends, and health staff.* Connection with support: *coping strategies.* Connection with peers: *sharing my experience.* | Valued friendship dimensions and actions  Cancer-related challenges to friendships |
| Sawyer et al. (2023), Canada | 12-22 | 8 | The experience of isolation in hospital and the impact on friendships for young people with cancer. | 1 in depth interview, 2 follow- up interviews | Transforming children and relationships | Cancer-related challenges to friendships  Changes to social needs, experiences, and outcomes |
| Schreiner (2020), USA | 14-20 | 28 | How friends support life after cancer. | Structured interviews | Living mindfully: *focusing on the present moment.* Living an identity as a healthy adolescent. Spending time with friends and family: *having emotionally deep conversations, engaging in outdoor activities with family and friends, developing, participating in, or reuniting relationships with friends.* | Valued friendship dimensions and actions |
| Sodergren et al. (2018), France, Israel, Norway, Poland, Netherlands and UK | 14-25 | 45 | Friend related quality of life. | Semi structured interviews | Social quality of life issues: *loss of friends, isolation from friends, opportunity to make new friends, appreciation of the support received from others.* | Valued friendship dimensions and actions  Cancer-related challenges to friendships |
| Stegenga (2014), USA | 14-17 | 10 | Impact of an event for young people with cancer to socialise and connect. | Semi structured interviews | Peer support. | Valued friendship dimensions and actions |
| Stegenga and Ward-Smith (2009), USA | 13-17 | 10 | Friends support at diagnosis. | Interviews guided by a global research question | Importance of friends and their reactions. | Valued friendship dimensions and actions  Changes to social needs, experiences, and outcomes |
| Thavakugathasalingam and Schwind (2022), Canada | 21 | 1 | Experience with friends during the cancer experience. | Narrative inquiry | Relationships *(isolated from friends).* | Cancer-related challenges to friendships |
| Walker et al. (2019), USA | 12-18 | 29 | Elements of life friends provide support for. | Semi structured interviews | It’s all good: *feeling supported moving forward.* My relationships: *being stronger and closer, having rocky friendships.* | Valued friendship dimensions and actions  Cancer-related challenges to friendships |
| Williamson et al. (2010), UK | 13-18 | 22 | Peers supporting YP with appearance changes. | Semi structured interviews (4, with photo prompts) and open-ended online survey (18) | Peer and family support: *my friends and family still tell me I am beautiful, peer shielding.* | Valued friendship dimensions and actions  Changes to social needs, experiences, and outcomes |
| Woodgate (2006), Canada | 12-18 | 15 | Experiences and examples of support from friends. | Semi structured interviews, moderate participant observations and focus groups | Supportive relationships: s*upport from my special friend.* Being there: *to comfort me, to keep me from feeling lonely.* Consequences of being there: *consequences associated with special friends.* | Valued friendship dimensions and actions  Changes to social needs, experiences, and outcomes |

Note: * = Inclusion criteria age range

# Table 2

## Characteristics of quantitative studies

| **First Author (Year), Country** | **Population** | | **Study topic** | **Method** | **Measures** | **Main findings** | **Code** |
| --- | --- | --- | --- | --- | --- | --- | --- |
|  | **Age range** | **Sample size** |  |  |  |  |  |
| Crowder et al. (2023), USA | 18-25* | 52 | Assessing relationships between social developmental factors and perceived social acceptance. | Observational | Attachment to parents and peers (IPPA), Peer relationship self-efficacy (ASSES), Social acceptance (SPPA) | Greater peer attachment was significantly related to greater peer relationship self-efficacy, higher perceived social acceptance, and higher perceived peer social acceptance. | Changes to social needs, experiences, and outcomes. |
| Ekim and Ocakci (2015), Turkey | 12-18 | 108 | Relationship between support and post-traumatic growth. | Observational | Social support (MSPSS); Posttraumatic growth (PGI) | Strong positive correlation (r = .73) between support from friends and posttraumatic growth. | Changes to social needs, experiences, and outcomes |
| Haluska et al. (2002), USA | 12-19 | 64 | Correlation between support from friends, family, total support and support satisfaction. | Quasi experimental with correlations reported | Social support: (SSQ, PSS-Fa, PSS-Fr) | No significant difference in social support from friends in YP with cancer and healthy YP. Perceived support from friends had a non-significant small positive correlation with total support (r = .21), and a significant small positive correlation support satisfaction (r= 0.28). | Changes to social needs, experiences, and outcomes |
| Hodges et al. (1984), USA | 12-18 | 20 | Exploring knowledge, feelings, attitudes, and reactions of class peers of adolescent cancer patients. | Observational | 50 items (true, false, or unsure): cancer knowledge, feelings, attitudes, and perceptions of patient and behaviours to help the patient. | Knowledge score mean of 4.70 (scale of 1-6). Of 5 one-way ANOVAs, only grade level had a significant effect on knowledge. Many were unsure of how the cancer peer may be feeling about their cancer, majority believe they/other classmates help patients but could do more to help. | Changes to social needs, experiences, and outcomes |
| Kay et al. (2019), USA | 12-24 | 115 | Impact of perceived support and conflict on psychological health. | Observational | Perceived support and conflict questions. Psychological distress (BSI-18), posttraumatic stress (PCL), positive affect (PANAS-C), posttraumatic growth (PTGI). | Support from friends significantly predicted psychological health (psychological distress, posttraumatic stress, positive affect, and post traumatic growth) in a hierarchical regression. | Changes to social needs, experiences, and outcomes |
| Manne & Miller  (1998), USA | 12-20 | 50 | Social support and psychological distress in YP with cancer. | Observational | Social support (NRI); Psychological distress (MHI-18) | Friends support and psychological distress had a non-significant, small correlation (r= -.22). Friends support and conflict were not significant predictors of psychological distress in a regression. | Changes to social needs, experiences, and outcomes |
| Tremolada (2016), Italy | 14-25 | 205 | Comparison, impact, and contributors of perceived social support. | Quasi experimental | HRQOL (MOS-SF-36); Social support (MSPSS) | Perceived social support from friends was significantly lower in young people with cancer than healthy peers (d= 0.21). Females, those with specific clinical profiles, and those with shorter time off-treatment had greater risk of poor social functioning. | Changes to social needs, experiences, and outcomes |
| Willard et al. (2020), USA | 13-23 | 192 | Perceived social functioning and social support | Observational | Self-perception (SPAA); Perceived social support (MSPSS); Affect (PANAS-C) | Those with lower self-perceived social functioning experienced significantly lower friend support compared to self-perceived average (p < .05) or high (p < .01) social functioning. | Changes to social needs, experiences, and outcomes |

Note: * = Inclusion criteria age range

IPPA: Inventory of parent and peer attachment (Armsden et al., 1987), ASSES: Adolescent Social Self-Efficacy Scale (Connolly, 1989), SPPA: Self-Perception Profile for Adolescents (Harter, 1988), MSPSS: Multidimensional Scale of Perceived Social Support (Zimet et al., 1990), PGI: Posttraumatic Growth Inventory (Tedeschi & Calhoun, 1996), SSQ: Social support questionnaire (Sarason et al., 1983), PSS-Fa: The Perceived Social Support From Family (Procidano & Heller, 1983), PSS-Fr: Perceived Social Support From Friends (Procidano & Heller, 1983), BSI-18: 18-item Brief Symptom Inventory (Derogatis, 2001), PCL: PTSD Checklist - Civilian Version (Weathers et al., 1993), PANAS-C: Positive and Negative Affect Scale for Children (Laurent et al., 1999), PTGI: 21-item Posttraumatic Growth Inventory (Tedeschi & Calhoun, 1996), NRI: Network of Relationships Inventory (Furman & Buhrmester, 1985) MHI-18: Mental Health Inventory-18 (Weinstein et al., 1989), MOS-SF-36: Medical Outcomes Study 36-Item Short-Form Health Survey (Reulen et al., 2006), SPAA: self-perception profile for adolescents (Harter 2012).

# Table 3

## Characteristics of mixed methods studies

| **First Author (Year), Country** | **Population** | | **Study topic** | **Methods** | **Main findings** | | **Code** |
| --- | --- | --- | --- | --- | --- | --- | --- |
|  | **Age range** | **Sample size** |  |  | **Quantitative** | **Qualitative** |  |
| Donovan et al. (2019), USA | 14-23 | 10 | Motivations to take part in online social support. | Singe-arm pilot.  Pre-test/post-test social support (PSS-Fr), psychosocial functioning (PCQL-32-PF), post-test open ended helpfulness question. | No significant differences from pre-test to post-test on measures of perceived social support. | Desire to connect with other cancer patients. | Valued friendship dimensions and actions  Changes to social needs, experiences, and outcomes |
| Dunsmore and Quine (1995), Australia | 15-24 | 51 | Information, support, and decision-making needs and preferences of young people with cancer. | Cross-sectional, exploratory, and descriptive. Survey with open- and closed-ended questions. | Preference to discuss cancer and treatment: another teenage with cancer 2nd (76%), a friend 4th (39%). Preference for support by talking about feelings or concerns: another teenager who had cancer 1st (59%), a friend 3rd (55%). Peer discussions rated as helpful (94%). Others who would benefit from information about cancer: friends who do not have cancer (72%). | | Changes to social needs, experiences, and outcomes |
| Enskar et al. (1997), Sweden | 20-23 | 10 | Impact of cancer on areas of life including friendships. | Semi structured interviews (guided by list of disease related problems). | 21 mentions of social problems related to their cancer. | Friends (as an area influencing the adolescents' experience of their life). | Changes to social needs, experiences, and outcomes |
| Steineck et al. (2022), USA | 14-25 | 32 | The role of digital technology in the lives of AYA living with advanced cancer. | Exploratory sequential design. Semi structured interviews followed by technology use categorisation to compare with demographic data. | Those that mentioned digital technology use were more likely to be male, older, and have relocated for treatment. | Maintaining existing peer support***:*** social media, video games, text messaging***.*** Connecting with peers with cancer: *text* message. | Valued friendship dimensions and actions |
| Valentino et al. (2023),  UK | 13-25 | 106 | Understanding young people’s experience in a peer program (Beads of Life). | Parallel design. Likert scale for program usefulness, enjoyment, and recommendation. Open ended questions. | Young people found the program useful, enjoyable, recommend to others. | Meeting others: *in the same boat, feel less alone, they understand better.* Sharing stories: *sharing experiences and hearing about others.* | Valued friendship dimensions and actions |
| Weidman et al (2022), Canada | 14-18 | 10 | Support experiences from friends, challenges, and desire to connect with cancer peers. | Explanatory sequential design. Survey and in person interview | 60% seek comfort from friends during difficult times. | Cancer journey difficulties: *related to friends/social network (n = 2).* Current support system: *friends (n = 7).* Perspectives on peer support (n = 8). | Valued friendship dimensions and actions  Cancer-related challenges to friendships |

*Only scales relevant to reported findings are included

PSS-Fr: Perceived social support friends (Procidano & Heller, 1983), PCQL-32-PF: Pediatric Cancer Quality of Life Inventory 32-Psychological Functioning subscale (Varni et al., 1998).

# Table 4

## Characteristics of resources for friends

| **Resource** | **Purpose** | **Development** | **Main content** |
| --- | --- | --- | --- |
| Masso (2011), USA.  Taking Charge: A cancer resource for friends. | Advice to help adolescents and young adults who may be unsure or scared support their friend with cancer. | Program Director American Childhood Cancer Organization | What to expect their friend may experience, ideas of how to help, communication tips, assessing online information, using social media or support, and ways to help increase awareness. |
| B-present (2022), USA.  Supporter Roadmap: Your unique path to connection and support after a cancer diagnosis. | Helping friends understand how to stay connected and provide meaningful support to a friend with cancer. | Created by cancer supporters and survivors with an understanding of the challenges supporters face. | Laying out steps to supporting a friend with cancer. Steps include understanding what their friend is facing, getting to know communication and support needs, creating a support group, emphasising commitment, and importance of self-care. Links to further resources on how to action some of these steps. |
| Wood and Canteen (2021), Australia. Wait… Did you say “cancer”: A guide to supporting your friend when they have cancer. | To provide advice for friends of young people with cancer. | In collaboration with young people with cancer. | Education about cancer and common questions (e.g. What is it like and the physical, life and emotional impacts). How can friends help including what to say, what not to say. Possible changes in friendships. A glossary and resources for where friends can get help. |
| Kavanaugh and The National Children's Cancer Society (2016), USA.  An Educational Guide for Friends of Teens with Cancer | To help young people who want to help a friend that has cancer and maintain friendship through a confusing and complicated time. | Written in collaboration with young people who have experience with cancer. | What cancer is, information on the experiences of young people with cancer, what young people with cancer appreciate and don't appreciate from their friends, and how to be a friend. |
| Lynda Jackson Macmillan Centre (2023), UK.  Young people with a relative or friend with cancer | To help young people who have a relative or friend with cancer to understand and cope with the situation. | Contribution from professionals, patients, and carers with expertise and experience in the area. | Coping with your own feelings, finding your own support, getting accurate information, and links to organisations and resources. |
| Rolfe and Queensland Youth Cancer Service (n.d.), Australia.  Supporting a Young Friend Through Cancer | Helping confused young people support a friend who has cancer. | Project Officer at Youth Cancer Services who support young cancer patients. | Common difficulties a young person with cancer faces, practical tips for how to help, and what to say or avoid saying. |
| Nemours Teen Health (2015), USA.  My friend has cancer. How can I help? | To help young people respond when a friend has cancer. | Medically reviewed by a doctor in youth cancer. | Physical and emotional changes to prepare for, ways to be emotionally supportive, specific practical support to offer, and taking care of yourself. |
| Ninox Cancer Support Crew (2022), Australia. The Shitshow Companion: How to be a good friend during cancer. | To educate young people who have a friend with cancer on what to say, what to do, or what support might be needed throughout and beyond treatment. | Includes insights from young people with cancer and their support network. | What is cancer and what it means, how to support and help a young person with cancer in various situations, how to support in the long-term. |
| Teenage Cancer Trust (n.d.), UK.  My friend has cancer. | To help young people know how to be there for a friend with cancer during and after treatment. | UK organisation offering support to young people with cancer. | What cancer is, common concerns a young person with cancer faces, how to help, what to avoid saying, practicing self-care and support for themselves. |
| Young Lives vs Cancer (n.d.), UK.  Be there for them: What you should do when your friend has cancer. | To help with young people be a good friend when they feel overwhelmed by a friend's cancer diagnosis. | Organisation supporting young people with cancer. | Information on treatment (where, how they may feel), how to help a friend feel connected, help during treatment, gift suggestions, helping after treatment, and supporting themself. |

**References**

An, H., & Lee, S. (2019). Difficulty in returning to school among adolescent leukemia survivors: A qualitative descriptive study. *European Journal of Oncology Nursing*, *38*, 70-75. <https://doi.org/10.1016/j.ejon.2018.12.008>

Arpaci, T., Altay, N., Yozgat, A. K., Yaralı, H. N., & Özbek, N. Y. (2022). ‘Trying to catch up with life’: The expectations and views of adolescent survivors of childhood acute lymphoblastic leukaemia about long‐term follow‐up care: A qualitative research. *European Journal of Cancer Care*, *31*(6), e13667. <https://doi.org/10.1111/ecc.13667>

B-present. (2022). *Supporter Roadmap: Your unique path to connection and support after a cancer diagnosis*. <https://b-present.org/young-adult-cancer-supporter-roadmap/>

Barton, K., Steineck, A, Walsh, CA, Lau, N, O'Donnell, MB, Rosenberg, AR. (2023). "I won't get to live my life the way I planned it": A qualitative analysis of the experiences of adolescents and young adults with advanced cancer. *Pediatric Blood and Cancer*, *70*(10), e30554. <https://doi.org/10.1002/pbc.30554>

Cassano, J., Nagel, K, O'Mara, L. (2008). Talking with others who "just know": Perceptions of adolescents with cancer who participate in a teen group. *Journal of Pediatric Oncology Nursing*, *25*(4), 193-199. <https://doi.org/10.1177/1043454208319972>

Cavusoglu, H. (2000). Problems related to the diagnosis and treatment of adolescents with leukemia. *Issues in Comprehensive Pediatric Nursing*, *23*(1), 15-26. <https://doi.org/10.1080/014608600265183>

Cheng, Y., Huang, C,, Wu, W., Chang, S, Lee-Hsieh, J, , & Liang, S., Cheng, S. (2016). The lived experiences of aboriginal adolescent survivors of childhood cancer during the recovering process in Taiwan: A descriptive qualitative research. *European Journal of Oncology Nursing*, *22*, 78-84. <https://doi.org/10.1016/j.ejon.2016.03.005>

Choquette, A., Rennick, JE, Lee, V. (2016). Back to school after cancer treatment: Making sense of the adolescent experience. *Cancer Nursing*, *39*(5), 393-401. <https://doi.org/10.1097/NCC.0000000000000301>

Crowder, S. L., Foster, R. H., Buro, A. W., Dillon, R., Godder, K., & Stern, M. (2023). Mediators of Social Acceptance Among Emerging Adult Survivors of Childhood Cancer. *Journal of Adolescent and Young Adult Oncology*. <https://doi.org/10.1089/jayao.2022.0120>

Daniels, S. R., Yang, C.-C., Toohey, S. J., & Willard, V. W. (2021). Perspectives on Social Media from Adolescents and Young Adults with Cancer. *Journal of pediatric oncology nursing : official journal of the Association of Pediatric Oncology Nurses*, *38*(4), 225-232. <https://doi.org/10.1177/1043454221992319>

David, C., Williamson, K., & Tilsley, D. W. (2012). A small scale, qualitative focus group to investigate the psychosocial support needs of teenage young adult cancer patients undergoing radiotherapy in Wales. *Supportive Care in Cancer*, *20*. <https://doi.org/10.1016/j.ejon.2011.08.002>

Donovan, E., Martin, S. R., Seidman, L. C., Zeltzer, L. K., Cousineau, T. M., Payne, L. A., Knoll, M., Weiman, M., & Federman, N. C. (2021). The role of social media in providing support from friends for adolescent and young adult (AYA) patients and survivors of sarcoma: Perspectives of AYA, parents, and providers. *Journal of Adolescent and Young Adult Oncology*, *10*(6). <https://doi.org/10.1089/jayao.2020.0200>

Donovan, E., Martin, S. R., Seidman, L. C., Zeltzer, L. K., Cousineau, T. M., Payne, L. A., Trant, M., Weiman, M., Knoll, M., & Federman, N. C. (2019). A Mobile-Based Mindfulness and Social Support Program for Adolescents and Young Adults With Sarcoma: Development and Pilot Testing. *JMIR mHealth and uHealth*, *7*(3), e10921. <https://doi.org/10.2196/10921>

Dunsmore, J., & Quine, S. (1995). Information, support, and decision-making needs and preferences of adolescents with cancer: Implications for health professionals. *JOURNAL OF PSYCHOSOCIAL ONCOLOGY*, *13*(4). <https://doi.org/10.1300/J077V13N04_03>

Ekim, A., & Ocakci, A. F. (2015). Relationship Between Posttraumatic Growth and Perceived Social Support for Adolescents With Cancer. *Journal of Hospice and Palliative Nursing*, *17*(5), 450-455. <https://doi.org/10.1097/NJH.0000000000000183>

Enskär, K., & Berterö, C. (2010). Young adult survivors of childhood cancer; experiences affecting self-image, relationships, and present life. *Cancer Nursing*, *33*(1), E18-24. <https://doi.org/10.1097/NCC.0b013e3181b6365a>

Enskar, K., Carlsson, M., Golsater, M., & Hamrin, E. (1997). Symptom distress and life situation in adolescents with cancer. *Cancer Nursing*, *20*(1), 23-33. <https://doi.org/10.1097/00002820-199702000-00004>

Fladeboe, K. M., Walker, A. J., Rosenberg, A. R., & Katz, L. F. (2021). Relationships between adolescents with cancer and healthy peers: A qualitative study. *Journal of Adolescent and Young Adult Oncology*, *10*(5). <https://doi.org/10.1089/jayao.2020.0133>

Haluska, H. B., Jessee, P. O., & Nagy, M. C. (2002). Sources of social support: Adolescents with cancer. *ONCOLOGY NURSING FORUM*, *29*(9), 1317-1324. <https://doi.org/10.1188/02.ONF.1317-1324>

Hodges, M. H., Graham-Pole, J., & Fong, M. L. (1984). Attitudes, knowledge, and behaviors of school peers of adolescent cancer patients. *JOURNAL OF PSYCHOSOCIAL ONCOLOGY*, *2*(2), 37-46. <https://doi.org/10.1300/J077v02n02_03>

Ingersgaard, M. V., Fridh, M. K., Thorsteinsson, T., Adamsen, L., Schmiegelow, K., &, & Baekgaard Larsen, H. (2021). A qualitative study of adolescent cancer survivors perspectives on social support from healthy peers-A RESPECT study. *Journal of Advanced Nursing*, *77*(4), 1911-1120. <https://doi.org/10.1111/jan.14732>

Kaluarachchi, T., McDonald, F., Patterson, P., & Newton-John, T. R. (2020). Being a teenager and cancer patient: What do adolescents and young adults with cancer find valuable and challenging with their friends and cancer peers? *Journal of Psychosocial Oncology 38*(2), 195-209. <https://doi.org/10.1080/07347332.2019.1672847>

Kavanaugh, B., & & The National Children's Cancer Society. (2016). *An Educational Guide for Friends of Teens with Cancer*. chrome-extension://efaidnbmnnnibpcajpcglclefindmkaj/<https://thenccs.org/wp-content/uploads/2021/09/education-guide-friends-teens-with-cancer.pdf>

Kay, J. S., Juth, V., Silver, R. C., & Sender, L. S. (2019). Support and conflict in relationships and psychological health in adolescents and young adults with cancer. *Journal of Health Psychology*, *24*(4). <https://doi.org/10.1177/1359105316676629>

Lam, C. G., Cohen, K. J., & Roter, D. L. (2013). Coping Needs in Adolescents with Cancer: A Participatory Study. *Journal of Adolescent and Young Adult Oncology*, *2*(1), 10-16. <https://doi.org/10.1089/jayao.2012.0011>

Larouche, S., & Chin-Peuckert, Lily. (2006). Changes in body image experienced by adolescents with cancer. *Journal of Pediatric Oncology Nursing*, *23*(4). <https://doi.org/10.1177/1043454206289756>

Lynda Jackson Macmillan Centre. (2023). *Young people with a relative or friend with cancer* chrome-extension://efaidnbmnnnibpcajpcglclefindmkaj/<https://www.ljmc.org/helpful_hints/hhc247_young_people.pdf>

Masso, A., & American Childhood Cancer Organization, . (2011). *Taking Charge: A Cancer Resource for Friends*. chrome-extension://efaidnbmnnnibpcajpcglclefindmkaj/<https://www.acco.org/wp-content/uploads/2014/12/Taking-Charge-Friends.pdf>

McDonnell, G. A., Shuk, E., & Ford, J. S. (2020). A qualitative study of adolescent and young adult cancer survivors' perceptions of family and peer support. *Journal of Health Psychology*, *25*(5). <https://doi.org/10.1177/1359105318769366>.

McLoone, J. K., Wakefield, C. E., Butow, P., Fleming, C., & Cohn, R. J. (2011). Returning to school after adolescent cancer: A qualitative examination of Australian survivors' and their families' perspectives. *Journal of Adolescent and Young Adult Oncology*, *1*(2). <https://doi.org/10.1089/jayao.2011.0006>

McNeil, R., Egsdal, M., Drew, S., McCarthy, M. C., & Sawyer, S. M. (2019). The changing nature of social support for adolescents and young adults with cancer. *European journal of oncology nursing : the official journal of European Oncology Nursing Society*, *43*. <https://doi.org/10.1016/j.ejon.2019.09.008>

Nemours Teen Health. (2015). *My Friend Has Cancer. How Can I Help?* <https://kidshealth.org/en/teens/friend-cancer.html>

Ninox Cancer Support Crew. (2022). *The Shitshow Companion: How to be a good friend during cancer.* . chrome-extension://efaidnbmnnnibpcajpcglclefindmkaj/<https://www.ninoxcsc.com.au/wp-content/uploads/2022/08/The-Shitshow-Companion.pdf>

Oh, S.-M., Lee, Hyejung., Kim, Sue., Kim, Sanghee., Lyu, Chuhl Joo. (2019). Social Adjustment of Adolescent Cancer Survivors: A Concept Analysis. *Child health nursing research*, *25*(3), 290-302. <https://doi.org/https://doi.org/10.4094/chnr.2019.25.3.290>

Olsen, P. R., & Harder, I. (2009). Keeping their world together: meanings and actions created through network-focused nursing in teenager and young adult cancer care. *Cancer Nursing*, *32*(6), 493-502. <https://doi.org/10.1097/NCC.0b013e3181b3857e>

Pini, S., Gardner, P., & Hugh-Jones, S. (2016). How teenagers continue school after a diagnosis of cancer: Experiences of young people and recommendations for practice. *Future Oncology*, *12*(24), 2785-2800. <https://doi.org/10.2217/fon-2016-0074>

Pini, S., Hugh-Jones, S., Shearsmith, L., & Gardner, P. (2019). 'What are you crying for? I don't even know you'-The experiences of teenagers communicating with their peers when returning to school. *European Journal of Oncology Nursing*, *39*, 28-34. <https://doi.org/10.1016/j.ejon.2018.12.010>

Pyke-Grimm, K. A., Franck, L. S., Halpern-Felsher, B., Goldsby, R. E., & Rehm, R. S. (2022). Day-to-Day Decision Making by Adolescents and Young Adults with Cancer. *Journal of pediatric hematology/oncology nursing*, *39*(5), 290-303. <https://doi.org/10.1177/27527530211068718>

Reuman, H., Kerr, K., Sidani, J., Felker, J., Escobar-Viera, C., Shensa, A., & Maurer, S. H. (2022). Living in an online world: Social media experiences of adolescents and young adults with cancer. *PEDIATRIC BLOOD & CANCER*, *69*(6), e29666. <https://doi.org/10.1002/pbc.29666>

Ritchie, M. A. (2001). Sources of emotional support for adolescents with cancer. *Journal of Pediatric Oncology Nursing*, *18*(3), 105-110. <https://doi.org/10.1177/104345420101800303>

Rolfe, M., & & Queensland Youth Cancer Service. (n.d.). *Supporting a young friend through cancer*. Queensland Government: Children’s Health Queensland Hospital and Health Service. Retrieved 5th May from

Salins, N., Muckaden, M. A., Ghoshal, A., & Jadhav, S. (2023). Experiences of Adolescents with Cancer Attending a Tertiary Care Cancer Centre: A Thematic Analysis. *Indian Journal of Palliative Care*, *28*(4), 428–433. <https://doi.org/https://doi.org/10.25259/IJPC_24_2022>

Sariman, J. A., Harris, N. M., Harvey, D., & and Sansom-Daly, U. M. (2022). The Experiences of Young People Living With Cancer in Regional and Remote Australia: A Qualitative Study. *Australian Social Work*, *75*(2), 205-218. <https://doi.org/https://dx.doi.org/10.1080/0312407X.2021.1977355>

Sawyer, J.-L., Mishna, F., Bouffet, E., Saini, M., & Zlotnik-Shaul, R. (2023). Bridging the Gap: Exploring the Impact of Hospital Isolation on Peer Relationships Among Children and Adolescents with a Malignant Brain Tumor. *Child & Adolescent Social Work Journal*, *40*(1), 91-105. <https://doi.org/10.1007/s10560-021-00764-x>

Schreiner, K., Grossoehme, D. H., Friebert, S., Baker, J. N., Needle, J. & Lyon, M. E. (2020). "Living life as if I never had cancer": A study of the meaning of living well in adolescents and young adults who have experienced cancer. *Pediatric Blood and Cancer*, *67*(10). <https://doi.org/10.1002/pbc.28599>

Sodergren, S. C., Husson, O., Rohde, G. E., Tomasewska, I. M., Vivat, B., Yarom, N., Griffiths, H., & Darlington, A.-S. (2018). A life put on pause: An exploration of the health-related quality of life issues relevant to adolescents and young adults with cancer. *Journal of Adolescent and Young Adult Oncology*, *7*(4), 453-464. <https://doi.org/10.1089/jayao.2017.0110>

Stegenga, K. (2014). Impact of a teen weekend on the social support needs of adolescents with cancer. *Journal of Pediatric Oncology Nursing*, *31*(5), 293-297. <https://doi.org/10.1177/1043454214531858>

Stegenga, K., & Ward-Smith, P. (2009). On receiving the diagnosis of cancer: The adolescent perspective. *Journal of Pediatric Oncology Nursing*, *26*(2), 75-80. <https://doi.org/10.1177/1043454208328767>

Steineck, A., Lau, N., Fladeboe, K. M., Walsh, C. A., Rosenberg, A. R., Yi-Frazier, J. P., & Barton, K. S. (2022). Seeking virtual support: Digital technology use in adolescent and young adults with advanced cancer. *PEDIATRIC BLOOD & CANCER*, *69*(11). <https://doi.org/https://dx.doi.org/10.1002/pbc.29938>

Teenage Cancer Trust. (n.d.). *My friend has cancer*. <https://www.teenagecancertrust.org/information-about-cancer/my-friend-has-cancer>

Thavakugathasalingam, M., & Schwind, J. K. (2022). Experience of childhood cancer: A narrative inquiry. *Journal for Specialists in Pediatric Nursing*, *27*(2), e12367. <https://doi.org/10.1111/jspn.12367>

Tremolada, M. a. B., Sabrina and Basso, Giuseppe and Pillon, Marta. (2016). Perceived social support and health-related quality of life in AYA cancer survivors and controls. *Psycho-Oncology*. <https://doi.org/10.1002/pon.4072>

Valentino, C., Daniilidi, X., & Portnoy, S. (2023). Evaluating the beads of life groups: hearing from our participants. *Clinical Child Psychology and Psychiatry*, *28*(1). <https://doi.org/https://doi.org/10.1177/13591045221090782>

Walker, A. J., Lewis, F. M., Lin, Y., Zahlis, E., & Rosenberg, A. R. (2019). Trying to feel normal again: Early survivorship for adolescent cancer survivors. *Cancer Nursing*, *42*(4), E11-E21. <https://doi.org/10.1097/NCC.0000000000000629>

Willard, V. W., Tillery, R., Gordon, M. L., Long, A., & Phipps, S. (2020). Profiles of perceived social functioning in adolescent and young adult survivors of childhood cancer. *Psycho-Oncology*, *29*(8), 1288-1295. <https://doi.org/10.1002/pon.5417>

Williamson, H., Harcourt, D., Halliwell, E., Frith, H., & Wallace, M. (2010). Adolescents' and parents' experiences of managing the psychosocial impact of appearance change during cancer treatment. *Journal of Pediatric Oncology Nursing*, *27*(3). <https://doi.org/10.1177/1043454209357923>

Wood, C., & Canteen. (2021). *Wait… Did you say “cancer”: A guide to supporting your friend when they have cancer.* chrome-extension://efaidnbmnnnibpcajpcglclefindmkaj/<https://www.canteen.org.au/sites/default/files/2022-12/Guide_to_supporting_friend_when_they_have_cancer.pdf>

Woodgate, R. L. (2006). The Importance of Being There: Perspectives of Social Support by Adolescents with Cancer. . *Journal of Pediatric Oncology Nursing*, *23(3)*, 122-134. <https://doi.org/10.1177/1043454206287396>

Young Lives vs Cancer. (n.d.). *Be there for them: What you should do when your friend had cancer.* . <https://www.younglivesvscancer.org.uk/life-with-cancer/my-friend-has-cancer/>
